# Social Entrepreneurship for Sexual Health (SESH): A New Approach for Enabling Delivery of Sexual Health Services among Most-at-Risk Populations

**DOI:** 10.1371/journal.pmed.1001266

**Published:** 2012-07-17

**Authors:** Joseph D. Tucker, Kevin A. Fenton, Robert Peckham, Rosanna W. Peeling

**Affiliations:** 1 University of North Carolina Project-China, Guangzhou, China; 2 Division of Infectious Diseases, University of North Carolina, Chapel Hill, North Carolina, United States of America; 3 Clinical Research Unit, London School of Hygiene & Tropical Medicine, London, United Kingdom; 4 Centre for Sexual Health and HIV Research, Research Department of Infection and Population Health, University College London, London, United Kingdom; 5 Centre for Humanities and Medicine, Li Ka Shing Faculty of Medicine, Hong Kong University, Hong Kong

## Abstract

Joseph Tucker and colleagues argue for social entrepreneurship, a new approach to help improve delivery of sexual health services.

**Summary Points**

- The dominant approach used to promote sexual health relies on centralized public clinic service delivery, unisectoral implementation, and vertically organized support (national/state/local public health structures).
- These systems have failed to test, link, and retain a large portion of most-at-risk populations.
- A social entrepreneurship for sexual health (SESH) approach focuses on decentralized community delivery, multisectoral networks, and horizontal collaboration (business, technology, and academia).
- Although SESH approaches have yet to be widely implemented, they show great promise. Social marketing and sales of point-of-care, community-based tests for HIV and other sexually transmitted diseases, conditional cash transfers to incentivize safe sex, and microenterprise among most-at-risk-populations are all SESH tools that can optimize the delivery of comprehensive sexual health interventions.

## Introduction

Each year there are over one billion estimated new curable sexually transmitted infections [1]. This is a daunting number, especially in the face of dwindling public health resources and difficulty reaching and retaining individuals in most-at-risk populations, who are the main drivers of these infections. Yet a growing number of community-based organizations focused on reaching most-at-risk populations have the capacity to move beyond condom distribution and conventional outreach to deliver novel point-of-care HIV/sexually transmitted disease (STD) testing [2], enhance partner notification [3], and link patients into treatment and care programs. The growing organizational and technical capacity of community-based organizations has been recognized by the Global Fund to Fight AIDS, Tuberculosis and Malaria, the GAVI Alliance, and the Joint United Nations Programme on HIV/AIDS, all of which have community-based organization representation at their highest levels [4]. But community-based organizations' growing capacity has yet to reach its full potential for service delivery since a substantial portion of most-at-risk populations in regions with substantial sexual disease burden remain out of care, untested, and unengaged [5].

Social entrepreneurship provides a new approach to more completely realize this full potential through identifying new prevention, treatment, and retention strategies. Optimizing health systems and program implementation are increasingly understood to be key drivers for improving health [6]. Social entrepreneurship uses entrepreneurial principles to promote the sustainable and innovative use of human, fiscal, and technological resources for social good. In the context of sexual health, social entrepreneurship focuses on developing novel, sustainable, community-responsive sexual health services. A number of social entrepreneurial tools, such as social marketing, conditional cash transfers, and microenterprise, have been effective in sexual health promotion in small pilot studies, but they have not been widely applied or systematically evaluated. Here we discuss the shortcomings of the dominant sexual health approach, explain the benefit of using social entrepreneurship for sexual health (SESH), and articulate key principles for moving forward.

## Current State of Sexual Health Service Delivery

The dominant approach for sexual health promotion is substantively and technically limited (Table 1) [7]. Sexual health services for most-at-risk populations are often guided by vertically organized public health/medical systems, ignoring the local horizontal partners (business experts, technology partners, academics, and others) that are necessary to fashion a sustained sexual health program [7]. The dominant approach prioritizes HIV prevention and treatment at the expense of syndemics (syphilis, human papillomavirus, and others) that are related to the same risky sexual behaviors [8].

**Table 1:** Overview of the dominant current sexual health delivery system and the SESH delivery system.

Variable	Dominant Sexual Health Delivery Approach^a^	SESH
Relationship to MARPs and CBOs	Limited engagement with CBOs serving MARPs	Authentic collaboration with formal steering input from CBO representatives
Time horizon	Short-term (e.g., 6–12 months) projects to promote MARP uptake	Middle- to long-term sustainable systems for ensuring MARP uptake
Financing	Mainly public funds	Public, private, public–private, and private–private investment structures
Implementation	Unisectoral, with vertical organizational links (national/state/local public health)	Multisectoral, with horizontal organizational links (academia, business, technology, etc.)
Clinical services	Formal, centralized clinic-based services	Decentralized CBOs, drop-in clinics, and mobile services in addition to clinic-based services
Substantive focus	HIV/STD-specific programs	Holistic sexual health focus
Comparative advantages	Organizational systems intact and widely used	Potential for innovative programs and sustainability
Comparative weaknesses	Sustaining local financing can be challenging, especially for stigmatized MARPs	Potential for miscommunication and poor governance to slow implementation

a The current sexual health delivery approach as exemplified by a public-sector STD service.

CBO, community-based organization; MARP, most-at-risk population.

In addition to a narrow substantive sexual health focus, the operational and implementation side of sexual health has also been narrowly conceived. Standard public health approaches administered by centralized public agencies remain the mainstay of HIV/STD services. This has created a roadblock for widespread implementation because ownership and engagement of most-at-risk populations in such approaches is often limited. While a broad range of community-based organizations have played key roles in advancing sexual health for most-at-risk populations, these organizations are only rarely involved in direct service delivery beyond testing. Furthermore, community-based organizations often rely on short-term and variable public-sector support.

## Social Entrepreneurship and Sexual Health

SESH challenges the dominant approach, drawing on the growing capacity of community-based organizations to advance new strategies and models for delivery of sexual health services (testing, linkage to care, and retention in care). Social entrepreneurship broadly defined is “the innovative use of resource combinations to pursue opportunities aiming at the creation of organizations and/or practices that yield and sustain social benefits" [9]. Although social entrepreneurship has advanced most rapidly in regions with an active civil society, social entrepreneurship has operated in a number of regions without a strong civil society.

The relationship of social entrepreneurship both to the global economic downturn and to revenue generation should also be clarified. Social entrepreneurship is not primarily focused on revenue generation [10], but rather is primarily about innovation and social change. While some social entrepreneurs will create mechanisms to effectively generate revenue, this is not a critical part of the strategic framework. Furthermore, while declining public health budgets in many local areas suggest the need for alternate resources, social entrepreneurship approaches are useful at any point in an economic cycle.

Social entrepreneurship has yet to be widely applied to the practice of promoting sexual health, but there have been small projects focused on social marketing of HIV/STD testing, conditional cash transfers, and microenterprise. Each of these tools demonstrates potential for SESH to optimize the delivery of high-quality sexual health services. The broader range of social entrepreneurship tools (social franchising, vouchers, and others) will not be discussed here, since their application to sexual health has not been well measured.

The principles of social marketing hold great promise for promoting condom use and HIV/STD testing. Marketing is “the activity, set of institutions, and processes for creating, communicating, delivering, and exchanging offerings that have value for customers, clients, partners, and society at large" [11]. Social marketing further refines this concept by focusing on marketing that adds social value. Social marketing identifies specific subgroups of most-at-risk populations, tailors messages appropriate for these subgroups, and conveys these messages via media and social networking capacities that are acceptable to the most-at-risk populations. Social marketing can make sexual health more attractive to subsets of high-risk individuals, incentivize healthy behaviors, and systematically reduce barriers associated with uptake of HIV/STD testing. Many condom promotion studies [12] and a few small pilot studies on HIV/STD testing [13] demonstrate the feasibility of social marketing to promote sexual health among most-at-risk populations. These pilot programs show how nuanced messages focused on subgroups of men who have sex with men (MSM) (e.g., young Latino MSM) can be more effective than generic MSM slogans. There are no rigorous studies evaluating how social marketing enhances detection of HIV-infected individuals in the population, or their retention in care [13].

Conditional cash transfers are another social entrepreneurship tool that could improve sexual health services. Conditional cash transfers are small sums of money given to poor households contingent on parents' investing in the health and/or education of their children [14], or small sums of money given to individuals who have negative STD tests [15]. Conditional cash transfers originated in Latin America during the 1980s, providing cash to families who ensured that their children went to school and attended regular health checkups. Conditional cash transfers work by increasing uptake of essential services among disadvantaged groups and accumulating human capital to break multigenerational cycles of poverty [16]. The underlying premise is that providing small financial incentives to reduce risky sexual behaviors can reap short-term and long-term behavioral change. A randomized study evaluating a conditional cash transfer program promoting school attendance among young women in Malawi showed decreased sexual activity among participants compared to controls [17]. Another randomized study in Tanzania providing small sums of cash to young people with negative STD test results found a 25% reduction in STDs associated with the intervention [15].

Microenterprise is another important tool for incentivizing uptake of sexual health services and behavior change. In the broadest sense, microenterprise is any small business. In the context of SESH, microenterprise empowers women and other vulnerable groups with skills training to decrease sexual risk. Given the known structural links between poverty and sexual risk, microenterprise has been extended to many women's groups in order to prevent HIV/STD infection and empower women [18]–[21]. Microenterprise could also take the form of community-based organizations directly selling rapid, point-of-care HIV and syphilis tests to most-at-risk populations. Two non-governmental organizations, Thailand's Population and Community Development Association [22] and mothers2mothers [23], have effectively used microenterprise for sexual health promotion. Microenterprise has been piloted among several groups of female sex workers: the women in the programs increased non-sex work employment [24], increased condom use [25], and had fewer sex work clients [26].

## A Tipping Point in Sexual Health Service Provision

Now is a “tipping point" in the evolution of sexual health service implementation. Several recent developments expand opportunities for social entrepreneurship in sexual health promotion: the transition from community-based organizations as prevention-oriented counseling services to service delivery organizations; the arrival of simple, user-friendly, point-of-care HIV/STD diagnostics on the global market; and the refinement of a substantial toolkit of evidence-based biomedical and behavioral health promotion measures. These developments provide the organizational locus (community-based organizations) and the substantive focus (novel testing and evidence-based interventions) to effectively use social entrepreneurship programs for sexual health promotion. These developments also suggest the settings where SESH tools could be most rapidly adopted—regions that have a range of multisectoral partners available and demonstrated sexual health needs. SESH is not a single “one size fits all" approach; it demands local input and community responsiveness to ensure success.

The growing capacity of community-based organizations to move beyond transient counseling and prevention activities to deliver sustainable, trusted, and culturally appropriate services demonstrates the advantages of this new approach. Local community-based organizations are often the laboratories for developing new solutions to complex sexual health problems, and their expanded scope in a number of countries sets the stage for a larger role in service delivery [2]. Social entrepreneur models have the potential to move beyond and extend the capacity of traditional community-organized HIV testing services. Social entrepreneur models are likely to be especially attractive to vulnerable groups compared to traditional community-based organization services for three reasons. First, social entrepreneurship models provide potential revenue sources and connections to marketing and business partners so that they can be sustained long term. Second, social entrepreneur models, especially if they are run as non-profit businesses by vulnerable groups for vulnerable groups, provide a deeper sense of ownership and greater ability to influence the design of innovative programs compared to traditional community-based organization programs. Finally, social entrepreneurship models represent an opportunity to more completely normalize the HIV testing process in culturally appropriate contexts. The expansion of public–private partnerships creates a nurturing environment to expand decentralized, sustainable systems for sexual health services [27].

Point-of-care HIV/STD diagnostics provide a new opportunity for social entrepreneurs of sexual health. While traditional public health programs have focused on placing these tests in clinics, a growing body of literature shows how these tests can be accurately and safely performed in non-clinical settings [28]–[30]. Social entrepreneurship can reconfigure financing and organizational systems to enhance point-of-care test uptake and linkage. For example, a community-based effort to expand point-of-care HIV testing among a subset of MSM could generate revenues that are reinvested into the program. Moving point-of-care diagnostics away from clinics and into non-governmental organizations, sex venues, and other informal settings will require guidance and input from a diverse group of individuals (public health leaders, technology experts, and business advisors) outside of the dominant approach. In addition to point-of-care HIV/STD diagnostics, we now have a robust toolkit of behavioral and biomedical interventions to prevent HIV/STD. From a biomedical perspective, antiretroviral therapy has emerged as a highly effective tool for primary HIV prevention [31]. This supplements other HIV prevention strategies that have shown effectiveness: antiretroviral therapy as pre-exposure prophylaxis, male circumcision, and prevention of mother-to-child transmission [32]. Among behavioral interventions, social-network-based condom promotion [33] and structural interventions [17] have both shown promise in randomized controlled trials.

## Key Principles for Implementation

Social entrepreneurship has the potential to create new models and strategies for improving sexual health among vulnerable groups at greatest risk for infection. In order to move this work forward, there are several key principles that can help guide implementation.

### Establishing Local Multisectoral Networks for Support and Linkage

Social entrepreneurship programs require the creation of multisectoral networks [34], including both local and regional networks to disrupt market forces that often limit scale-up. A multisectoral approach incorporates a number of unique partners, each with distinct contributions that are essential for effective social entrepreneurship (Table 2). Although social entrepreneurial programs can be designed, implemented, and evaluated by a single organization, having local networks catalyzes this process and increases the likelihood of sustainability. Business and marketing expertise can be invaluable for effectively designing campaigns to promote sexual health service utilization among subsets of most-at-risk populations. Clinical partnerships are also critical because individuals who access community-based services must be linked and retained in clinical care. In addition to business and medical partners, incorporating expertise on the legal and regulatory framework of sexual health (point-of-care testing regulations, etc.) is also important.

**Table 2:** Key partners in a multisectoral SESH program.

Local Partner	Potential Contribution	Limitations
**Government public health bureau**	-Identify high-risk groups, venues	-Lack of trust among vulnerable groups in some local contexts
	-May coordinate the multisectoral response in many contexts	-Often have limited interactions with other sectors in sexual health programming
	-Capacity to coordinate with other government agencies to form a response	
	-Potential for influencing policy	
**Business/marketing**	-Provide consultation on social marketing, microfinance	-Many local organizations have limited human personnel to implement business programs
	-Advise on strategic planning and market analysis for point-of-care tests	-Double bottom line initiatives are often markedly different from traditional business practice
	-May coordinate the multisectoral response in some contexts	
**Technology and laboratory science**	-Provide point-of-care test monitoring and evaluation	-Broader social context of technology and its use are frequently overlooked
	-Recommendations on using specific point-of-care tests	
**Social change programs**	-Adopt sexual health programs into popular, ongoing social change programs	-May be challenging to incorporate in some regions
**Academic institutions**	-Conduct research and evaluate whether programs are effective	-Lack of trust among vulnerable groups in some local contexts
	-May coordinate the multisectoral response in some contexts	-Often have limited interactions with other sectors in sexual health programming
**Clinical medicine**	-Provide high-quality preventive and therapeutic clinical services	-Often have limited interactions with other sectors in sexual health programming
	-Link and retain most-at-risk populations within traditional clinical services	
**Law**	-Assist with the formation and development of formal and informal non-governmental organizations	-Limited experience focused on sexual health
	-Help identify and overcome regulatory barriers for community service delivery	

### Using Entrepreneurial Principles and Organizations to Promote Innovation

There are several mechanisms whereby social entrepreneurship fosters innovation. First, multisectoral networks cross disciplinary and sectoral boundaries in order to encourage broader thinking about sexual health services. Formally incorporating communications, business, and other partners can spur new thinking about old sexual health problems. Second, conventional sexual health service provision assumes that most-at-risk populations will not and cannot afford to pay for services. An entrepreneurship model challenges this assumption, creating an opportunity to fundamentally reconsider financing systems. Finally, the social entrepreneurship movement has spawned a number of local and global organizations intended to promote the practice of social entrepreneurship [35]. These established incubators can help individual groups focused on creating innovative sexual health services.

### Enhancing Health Impact through Wider Access to New Technology

Achieving the population-level benefits of high-quality sexual health services requires that essential diagnostic technologies move beyond the laboratory and the clinic. Simple, rapid STD tests that do not require reagents or trained personnel are now commercially available for syphilis, chlamydia, and gonorrhea. The World Health Organization bulk procurement scheme ensures low prices in many low-income states, so that this new technology can be more rapidly scaled up [36]. Social entrepreneurship provides the organizational, financial, and social basis for more completely taking advantage of these new point-of-care HIV/STD tests.

### Improving Engagement of Most-at-Risk Populations and Accountability in Service Provision

Focusing on needs and services identified for most-at-risk populations is essential for effectively implementing a SESH approach. Community-based organizations serving most-at-risk populations in many regions are increasingly capable of providing point-of-care HIV/STD testing and associated sexual health services [2]. The dominant public health approach allows only token input from community-based groups and, not surprisingly, results in unbalanced relationships between community groups and the public health system. These relationships need to be re-balanced to recognize the growing organizational and technical capacity of community-based organizations.

### Focusing on Holistic Sexual Health Services instead of Narrow Disease-Specific Strategies

While HIV control has spurred a number of major advances in sexual health, a broader focus on sexual health is both more responsive to the needs of individual most-at-risk populations and more likely to be sustained long term. Integration of disease-specific programs into more holistic sexual health care provision has been shown to be effective in many settings [37], including STD clinics [38]. Furthermore, emphasis on wellness and prevention may be more effective than disease-focused treatment among some most-at-risk populations [39].

### Evaluating and Ensuring That Learning Iteratively Improves Service Delivery

The effectiveness of traditional sexual health campaigns is measured in terms of health outcomes, but the incorporation of entrepreneurial methods requires new metrics. Double bottom line projects measure both health and entrepreneurial outcomes, which can be measured in several ways (for more information, see the Research Initiative on Social Entrepreneurship [http://www.riseproject.org/]). These metrics are capable of evaluating the process, outcomes, and monetization of both non-profit and for-profit endeavors. One example of a social entrepreneurship metric is the “balanced scorecard," a tool that measures operational performance in terms of financial, customer, business process, and learning-and-growth outcomes. The findings of evaluations must be used to iteratively improve the SESH approach.

## Potential Challenges to SESH

The SESH paradigm must be critically scrutinized to understand how it can be locally adapted, scaled up, and monitored. There will be challenges in applying this framework, including overcoming hesitation about commercializing sexual health and identifying donors and business partners willing to collaborate with stigmatized groups. Expanding training and capacity building among community-based organizations will be critical for ensuring implementation [2]. Strong local networks that connect medical/public health structures and community-based service providers are also key linkages for achieving health outcomes. Local community-based organizations that use a SESH approach will require governance structures and transparency [2] to ensure that revenues are reinvested in direct service provision. A SESH approach will not be sustainable without careful financial planning and the capacity to offer trusted, comprehensive, and highly valued sexual health services. Effectively communicating the meaning and value of social entrepreneurship as it applies to sexual health is also important, since social entrepreneurship is a relatively new concept [34]. Finally, there are legal and regulatory hurdles in sexual health service provision [36] that will need to be identified and overcome for SESH to become a powerful systems paradigm.

## Conclusions

There will be no magic bullet in responding to global sexual health crises, but delivery systems are clearly shifting as the global economic crisis continues. Donor contributions to the national sexual health services of low-income countries are shrinking, and the sexual health budgets of high-income countries are similarly strapped. International funding for HIV programs has fallen from US$8.7 billion to US$7.6 billion, and the Global Fund to Fight AIDS, Tuberculosis and Malaria announced there would be no new programs until 2014 [5]. Increasingly limited public resources, alongside persistent demand for high-quality sexual health services, require a reconsideration of strategies and innovative models for delivery. New point-of-care testing technology and increased community-based organization capacity suggest how a SESH approach could accelerate sexual health testing, linkage, and retention in care. The SESH approach will not replace the dominant service delivery system, but may prove effective in reaching and sustaining engagement with individuals who may be impossible to reach using a conventional delivery system. The global economic crisis has already forced many community-based organizations to rethink their financial model and cut back services, but SESH tools may provide a pathway to sustainable and effective delivery of sexual health services.

## Abbreviations

MSM: men who have sex with men
SESH: social entrepreneurship for sexual health
STD: sexually transmitted disease
